# Connecting concrete technology and machine learning: proposal for application of ANNs and CNT/concrete composites in structural health monitoring

**DOI:** 10.1039/d0ra03450a

**Published:** 2020-06-17

**Authors:** Sofija Kekez, Jan Kubica

**Affiliations:** Silesian University of Technology Akademicka 2A 44-100 Gliwice Poland sofija.kekez@polsl.pl

## Abstract

Carbon nanotube/concrete composite possesses piezoresistivity *i.e.* self-sensing capability of concrete structures even in large scale. By incorporating smart materials in the structural health monitoring systems the issue of incompatibility between monitored structure and the sensor is surpassed since the concrete element fulfills both functions. Machine learning is an attractive tool to reduce model complexity, so artificial neural networks have been successfully used for a variety of applications including structural analysis and materials science. The idea of using smart materials can become more attractive by building a neural network able to predict properties of the specific nanomodified concrete, making it more cost-friendly and open for unexperienced engineers. This paper reviews previous research work which is exploring the properties of CNTs and their influence on concrete, and the use of artificial neural networks in concrete technology and structural health monitoring. Mix design of CNT/concrete composite materials combined with the application of precisely trained artificial neural networks represents a new direction in the evolution of structural health monitoring of concrete structures.

## Abstract 2

Carbon nanotube/concrete composite possesses piezoresistivity *i.e.* self-sensing capability of concrete structures even in large scale.

## Graphical abstract

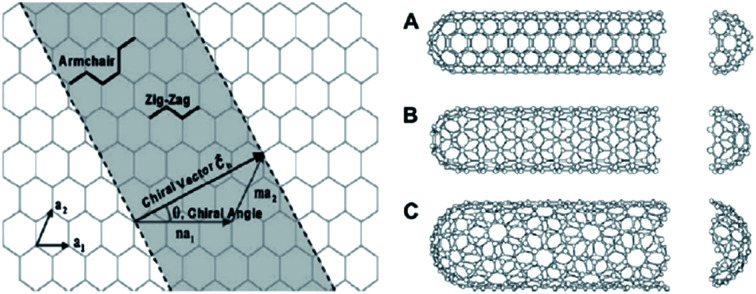

## Introduction

Both nanotechnology and neural networks were in the making during the first half of the twentieth century. The first idea of nanotechnology from R. Feynman (Caltech, 1959) and the first stage of development of artificial intelligence by B. Widrow and M. Hoff (Stanford, 1959) marked the beginning of a new era of technology. The conference held in Trieste, Italy in 1984 (ref. 1) opened a discussion on the topic of transforming brain functions onto a mathematical level. Johannesma introduced concepts for the analysis of neural responses to external stimuli, which would be used for an objective definition of receptive fields. However, the development was gradual until Ng and Dean created a network that learned to recognize a high-level concept (“cat or no cat”) in 2012. Extensive work followed, making machine learning technology approachable and usable in many different areas. Artificial neural networks (ANNs) were primarily developed as a representation of a human brain, however over time it became a tool for solving specific tasks. On the other hand, nanotechnology has remained theoretical and was facing a halt until the discovery of fullerenes in 1985 and carbon nanotubes (CNTs) in 1991 by Sumio Iijima. The initial tests showed that mechanical properties of carbon nanomaterials are superior to the materials which were used at that time. This was the main reason for various investigations of their properties since, and for many fields of engineering and medicine to establish a way to apply carbon nanomaterials.

As for civil engineering, it wasn't until the brink of 21^st^ century that carbon nanomaterials found their use. Even though concrete is a conventional material, it is the most used material in the world after water as humanity's first and basic need. Suffice to say that abundant funds are invested every year in concrete structures and their wellbeing. Every civil structure is designed to meet the criteria of safety, durability, serviceability, and sustainability during its service life so constant supervision over its condition is necessary. For exactly that purpose structural health monitoring (SHM) was developed as a field which deals exclusively with optimizing the structure's behavior during its exploitation period.

SHM has now grown into a highly developed field in terms of new and emerging technologies. The most recent developments are connected to different types of nanosensors^2,3^ such as transducers,^4^ electrochemical and optical^5^ sensors as well as smart materials. While the development of nanoscale devices and nanoelectromechanical systems utilizing the unique properties of carbon nanotubes (CNTs) is an evolving area of nanotechnology, there is also considerable interest in making macroscopic engineered, smart, materials that can exploit novel material properties.^6^ The existing problem with monitoring devices has been the compatibility between the sensor and the concrete structure. Smart materials with the addition of carbon nanomaterials are being developed in order to surpass this issue of incompatibility by making the whole structure able to give feedback about its condition. Cement-based materials with the addition of CNTs manifest piezoresistive behavior, meaning that it is possible to monitor strain by following the change in the electrical resistance^7^ under certain type of physical stimuli affecting the structure. This way, structural elements not only fulfill a structural function^8^ but also behave as sensors apt for self-monitoring, while the data acquired and collected is regarded as usable.

Machine learning has been used in the field of concrete technology in the past twenty years. Various neural networks were made^9–26^ in order to predict primarily the compressive strength of concrete with different additions. Nonetheless, ANNs may be used also for predicting electrical conductivity of smart concrete, similar to the model used for conductive nanomodified polymer materials.^132,144^ The idea of using smart materials can become more attractive by building a neural network able to predict properties of the specific nanomodified concrete, making it more cost-friendly and open for unexperienced engineers.

This paper is organized as follows. Section 2 and 3 give an overview of the properties of carbon nanotubes and further focus on the electrical properties of CNT/concrete composite materials. Section 4 establishes the role of artificial neural networks in engineering and their application. Section 5 gives a review on the structural health monitoring and state-of-the-art in this field and proposes a new method of designing smart materials capable of self-sensing.

## Electromechanical properties of CNTs

### Physical properties of CNTs

Since Iijima *et al.*^27^ described “single-shell tubules” three decades ago, there has been extensive research on this topic and many discoveries have been made regarding the properties and possible applications of single-walled carbon nanotubes. Carbon nanotubes are allotropes of carbon with a cylindrical structure. As a part of the fullerene family, nanotubes are members which have a long hollow structure made of one or more sheets one carbon atom thick. The sheet rolling angles are chiral and the radius of the tube determines the properties of the carbon nanotube.^28–30^ Some of the highlighted characteristics of CNTs that are superior to other materials are Young's modulus,^5,30–35^ tensile strength,^31,32,34–36^ mass density,^4,35,37^ as well as electrical^38–40^ and thermal conductivity (Table 1). These properties offer CNTs great potential for wide applications in field emission, conducting plastics, thermal conductors, energy storage, conductive adhesives, thermal interface materials, structural materials,^40^ fibers, *etc.*^34^

**Table 1:** Properties of concrete and reinforcing steel (C 90/105, S 600-highest classes according to Eurocode), and CNT (values collected from ref. 4, 5 and 30–41)

Material	Tensile strength [MPa]	Young's modulus [GPa]	Mass density [g cm^−3^]	El. conductivity [S m^−1^]
C 90/105	5	44	2.5	10^−8^
S 600	600	210	8.75	1.45 × 10^6^
CNT	100 000	1000	1.6	10^7^

**Fig. 1:**
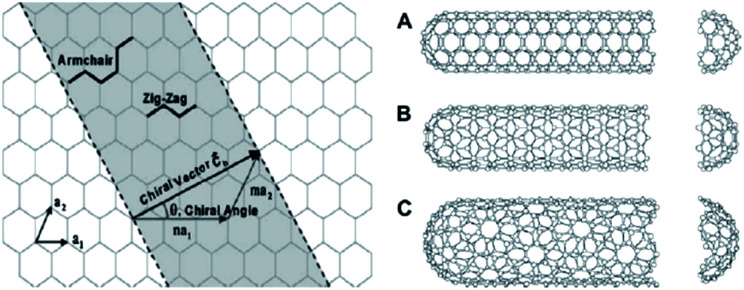
Schematic of how a sheet of graphene is rolled to form a CNT with different chiralities, (A) armchair; (B) zigzag; (C) chiral.^35^

The electrical conductivity of an individual CNT is quite high, however, the electrical properties and performance of CNTs depend on several geometric and chemical factors describing a single tube, as well as subsequent treatment to which it is exposed. Some analytical approaches for determination of the electrical conductivity are based on the continuum percolation theory of 3D cylinders,^42^ where CNTs were modeled with finite length and diameter.^43^ The nanotube electronic property is a strong function of its atomic structure, mechanical deformation, and chemical doping.^34,44^ Nanotubes can be electrically conductive or semiconductive,^4–6,34,45–47^ depending on the chirality angle (Fig. 1) and uniformity after production.^48^ Chirality determines the configuration of stacked atoms in the carbon sheet, meaning that if the CNT is chiral *i.e.* cannot match its mirror image, conductivity needs to be additionally determined, especially for nanotubes with relatively small radii.^49^ As Wang^50^ concluded after examining orthogonal motion transmission between aligned CNTs, chirality directly affects motion-transmission factors denoting the effect on mechanical power transmission in nanomaterials with extreme surface-to-volume ratio. Joshi *et al.*^51^ examined the influence of chirality on the dynamic behavior of single-walled CNTs. The simulation was made for cantilever nanotube considering it as a space frame structure similar to 3D beams with point masses. The results indicated that the existence of defects, and not chirality, affects the bending rigidity of the SWCNT, which gives valuable information about the nature of the influence of chirality to the behavior of nanotubes.

### Actuation behavior

Actuation behavior of nanotubes was first reported by Baughman *et al.*,^52^ showing that the mechanical deformation of nanotubes appears as a result of the injection of electrical charge. The demonstration of electrochemical actuation of CNTs was made using a single-walled buckypaper working as an electrode of an electrochemical cell. It was concluded that the classical electrostatics is also applicable at the nanoscale,^52,53^ opening a new topic and triggering researchers to further examine this occurrence. Later, Li *et al.*^49,53,120^ wrote on the behavior of CNTs exposed to an electrical charge. The authors found that the electrical failure of nanotube depends on the level of charge as well as its length. Many types of research have been conducted on the topic of nanotube characteristics and behavior, giving guidelines for further examinations. What is undoubted is that CNTs represent building blocks^54^ for any kind of materials or devices where conductance is paramount. It is indeed this combination of mechanical and electrical properties of individual nanotubes that makes them the ideal reinforcing agents in a number of applications.^4^

## CNT/concrete composite

### Influence of conductive filaments on concrete

Concrete is a nanostructured, multi-phase, composite material that ages over time. It is composed of an amorphous phase, crystals, and bound water. The amorphous phase, calcium–silicate–hydrate (C–S–H) is the ‘glue’ that holds the concrete together and is itself a nanomaterial.^55–58^ The properties of concrete exist in multiple length scales (from nano to micro to macro) where the properties of each scale derive from those of the next smaller scale *i.e.* processes occurring at the nanoscale ultimately affect the performance of the bulk material.^55,59–67^ Viewed from the bottom up, concrete is a composite of molecular assemblages, surfaces, and chemical bonds that interact through chemical reactions, intermolecular forces and diffusion. The combination of exceptional mechanical properties of CNTs along with their low density and one-dimensional structure makes them an exceptional candidate for reinforcement in composite materials.^35,55,59^

**Fig. 2:**
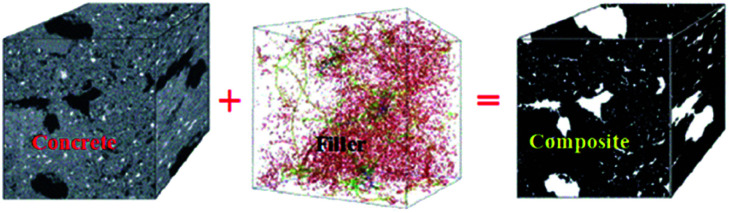
Structure of concrete with functional filler.^39^

Incorporating some functional materials (Fig. 2) such as carbon nanotubes or carbon fibers into conventional concrete gives it the ability to sense strain, stress, cracks or another type of damage while maintaining or improving its mechanical properties and durability.^40,56,57,68–72^ When added to concrete, CNTs work as fillers in the mesoporous environment of concrete, improving the hydration of cement and decreasing porosity, thus improving the compressive strength and decreasing autogenous shrinkage of concrete. Nanoscale reinforcement can also inhibit crack growth in the initial stages of damage and so prevent damage propagation, enhance the quality of the paste–aggregate interface and increase the amount of high stiffness C–S–H.^35,36,59,72–74^ Li *et al.*^75^ investigated the mechanical properties of CNT/cement composites and found that compressive and flexural strengths of cement with 0.5 wt% CNT addition were increased by 19% and 25%, respectively, compared to the plain unreinforced cement.^48^

Fu and Chung^76^ have researched the strain sensitivity of carbon fiber-reinforced cement compared to that of normal cement since 1993. Furthermore, Thostenson and Chou^49,77^ proposed that the change in the size of reinforcement, from micron-sized fiber reinforcement to carbon nanotubes with nanometer-level diameters, enables the opportunity for multi-functional sensing capability. By *in situ* combining reinforcements of different scales, carbon nanotubes can penetrate the matrix-rich areas between fibers in individual bundles as well as between adjacent plies and can achieve a nerve-like network of sensors throughout the arrays of fibers in a composite. More studies evaluated the influence of hybrid reinforcement, such as a combination of carbon fiber (CF) and carbon black with CNTs on the self-sensing properties of cement-based sensors.^25,40,72,78–81^ Anthony *et al.*^80^ developed computational models of a composite for predicting the benefits of grafting CNTs at the CF surface in order to create a conductive network that can be used for *in situ* damage detection. Moreover, Han *et al.*^81^ showed that the flexural strength and electrical conductivity in the cement mortar matrix with self-assembled CNT/nano carbon black composite fillers are enhanced and stable, and sensitive piezoresistivity is achieved at a low filler content. Since the fillers are mainly in order of magnitude of microscale or nanoscale, the potential filler–matrix and filler–filler interface surface areas of the composite are enormous. Since these interfaces have an effect on the quality of the electrical communication between CNTs and concrete matrix and also on communication between adjacent CNTs, they are affecting the conductive network and the overall electrical conductivity.^56^

### Dispersion of CNTs

The mechanical properties and electrical conductivity of concrete/CNT composite depend on the dispersion state of the nanofillers. The nature of the dispersion problem for CNTs is somewhat particular because of their small diameter in the nanometer scale with a high aspect ratio (>1000)^4,48,72,82–84^ resulting in an extremely large surface area. The aspect ratio must be sufficiently large to maximize the load transfer between the CNTs and matrix material and thus to achieve enhanced mechanical properties.^4,35,85^ Proper dispersion of the nanoparticles in concrete or cement mortar matrix is vital for improvements in the mechanical performance of the composite but it is difficult to achieve for nanomaterials with a large aspect ratio such as CNTs due to their high surface energy and strong interparticle (van der Waals) forces. If not properly dispersed, the addition of nanomaterials to cementitious materials may finally result in a decline in the mechanical properties.^35,36,43,59,73,81,86^ It has been found that CNTs tend to dramatically hamper the mechanical properties of fabricated nanocomposites.^4^

Namely, the one-dimensional fillers are more difficult to disperse and easier to damage during the fabrication of concrete. As the surface area of the filler increases, the attraction forces between the fillers also increase.^56,87^ Especially for fibrous fillers, high aspect ratios combined with high flexibilities increase the possibility of fiber entanglement and close packing.^81^ Studies have emphasized repeatedly^74,88–90^ that CNTs tend to agglomerate, which hinders their uniform distribution within a matrix.^91^ Poor dispersion of nanoparticles may lead to segregation, weak zones or potential areas for aggregation of concentrated stresses.^36,86^ As an alternative, Anthony *et al.*^80^ showed how CNTs can be directly grafted on carbon fibers in order to form a hierarchical reinforcement, combining nanoscale and microscale reinforcements^92,93^ and avoid such dispersing problems. Uniform dispersion can be achieved using various types of mechanical and chemical methods including ultrasonication, shear mixing, calendaring, ball milling, stirring and extrusion. Various methods of chemical modification have been proved quite successful in contributing to better nanotube dispersion. In terms of tensile modulus, it has been established by numerous studies^94,95^ that chemically modified nanotubes exhibit a significant increase as compared to the matrix resin. This is mainly due to the fact that functionalization improves both dispersion and stress transfer.^82^ Some studies have proved that the carboxyl CNT^41^ is much easier to disperse in water and concrete matrix than the plain CNT because of the carboxyl groups situated on the surface of the carbon sheet. Moreover, the carboxyl CNT achieves a better bond with the concrete matrix than the plain CNT.^41^ Surely that the selection of a proper method or a combination of several methods and their processing conditions need to be based on the desired properties of the end product.^35^

### The sensing ability of CNT/concrete composite

Concrete has a volume conductivity lower than 10^−10^ S cm^−1^ (ref. 35) which makes it an electrically nonconductive material.^40^ However, the addition of CNTs provides it with new properties^2,56,96^ making it a multifunctional structural material, primarily because the CNT/cement composites display piezoelectric behavior.^36,43,97–99^ Since first-ever CNT/polymer composites were reported in 1994 by Ajayan *et al.*,^100^ many authors^7,82,101–104^ showed how the conductive carbon nanofillers such as CNTs can form conductive networks in polymeric materials at extremely low weight fractions while simultaneously improving the fracture toughness. Namely, conductive nanofillers were found to impart significant conductive and piezoresistive behavior to polymers making such material systems feasible for electrical applications, such as strain sensors.^8,79,105^ The same principle can be applied to concrete which similarly represents an insulating matrix.^57,74^ By measuring the changes in electrical resistance of the CNT/concrete composite material under certain strain, its structural health can be monitored in terms of stress, strain, and damage.^41^ Saafi^97^ presented CNT networks that were embedded into a cement matrix with the idea to develop an *in situ* wireless and an embedded sensor for damage detection in concrete structures. The results showed that the wireless response of the sensors changed due to the formation of cracks during loading and that the CNT/concrete sensors were able to detect the initiation of damage even at an early stage of loading. Sett^106^ first fabricated beams and rings using the polymer concrete with the CF filler. He tested the relationship between the change in electrical resistance and loading as well as the relationship between resistance change and deflection of the beams under four-point bending.^41^

#### Percolation theory

Concrete cannot be used for sensor purposes in its basic form, but if a metallic material is dispersed into it a conductive composite can be manufactured. Moreover, a non-conductive or poorly conductive matrix has greater sensitivity to changes in electrical resistance^40^ which makes this combination theoretically perfect. At low volume fractions,^49^ the conductivity of the composite remains very close to the conductivity of the pure insulating matrix. However, when a certain volume fraction is reached, the overall electrical conductivity experiences a drastic increase by many orders of magnitude^31,72^ due to the formation of continuous electron paths or conducting networks. This phenomenon is known as percolation and the critical concentration of the filler as percolation threshold.^41,57,107^ Numerous experimental and theoretical studies agree that the electrical conductivity of CNT reinforced cement-based composites is governed by a percolation process.^107^ It is well known that the percolation is associated with the ability of electron transfer along CNTs as well as between adjacent nanotubes at their junctions.^43^ Hence, the percolation theory is applied to explain the electrically conducting behavior of composites consisting of conducting filler and insulating matrices.^105,108–112^ Below the percolation transition range, there are no electron paths, the electrical properties are dominated by the matrix material^35,56^ and there is no conductivity achieved.^8,107^ Small size and large aspect ratio of CNTs grants them an exceptionally low percolation threshold meaning that the volume fraction for achieving conductivity is satisfactorily low.^72,113–115^ Pan *et al.*^116^ modelled the CNTs within percolation networks as ellipsoidal inclusions uniformly distributed in 3D space ‘assuming contacts at the CNT junctions with zero resistance’, and other calculations of the electrical conductivity were conducted using a similar micromechanics model.^117^ It can be concluded that the level of distribution within the concrete/CNT composite is threefold: (i) the distribution of functional filler in a binder, (ii) distribution of the binder with fillers among fine aggregates, and (iii) distribution of the fine aggregates with binder and fillers among coarse aggregates.^41,118^ As previously stated, the potential filler–matrix and filler–filler interfaces affect electrical bonds between fillers as well as between the concrete matrix and the filler material,^119^ thereby influencing the comprehensive conductive network and electrical conductivity of the composite.^41,56^

#### Piezoresistive behavior

Carbon nanotubes express interesting electromechanical properties. When subjected to stress, their electrical properties change indicating a linear and reversible piezoresistive response with respect to different stress levels.^3,48,79,121–124^ Han *et al.*^41^ manufactured a concrete/CNT composite with a compressive strength of about 40 MPa. It was proven that electrical conductivity strongly depends on the morphology of the CNT network^56,92^*i.e.* the number of contact points and that the electrical resistance of self-sensing CNT concrete can be changed when it is deformed under applied loading. Han *et al.*^41^ showed that the changes in resistance coming from the alteration of nanotubes' geometry under external loading may be neglected since they are small and reversible. However, the contact resistance must be taken into consideration since the distance between adjacent nanotubes may considerably differ when under load, implying the sensing ability is stable and reversible as long as the deformation is elastic.^40,41^

Piezoresistivity of composites with conductive fillers has been the subject of several numerical and analytical works,^105,125,126^ and simulations of the processes taking place in these materials are taking a lot of interest. Most recently, the authors^107^ presented a 3D generalization of the micromechanics model of the overall conductivity and uniaxial piezoresistivity of CNT-reinforced composites subjected to arbitrary strain states. Changes in conductance, as the origin of the piezoresistive behavior, expected when the CNT/concrete composite is subjected to external mechanical strains^105,107,127–129^ are here prescribed to the changes in the volume fraction, to filler reorientation, and to changes in the inter-particle properties. The authors^107^ proposed an approach shown to be capable of determining the piezoresistivity coefficients by two virtual tests, namely laterally constrained dilation and distortion, which is efficient in terms of computational cost and, more importantly, it reliably presents the complex electromechanical behavior of CNT/concrete composites. Piezoresistive behavior of CNT/concrete composites is continuously investigated because it is the base point on which the majority of sensoring systems used in SHM today were built on.

## Artificial neural networks

### Development of artificial neural networks

Based on Hebbian learning, as it is conceived on principles of the biological systems' dynamics, artificial neural networks (ANNs) have been developed as an interconnected group of nodes inspired by a simplified representation of neurons in the brain. According to Braitenberg (1977), changing the stimuli conditions may induce clusters to form and later “evaporate” in order to build new configurations, more or less mimicking the way the corresponding neurons are involved in a dynamic succession of different assemblies, “ignited” by the changes in the external world.^1^ Although the original idea of ANNs was to solve problems in the same way as a human brain would, over time it deviated from biology and advanced towards the direction of solving specific tasks (pattern recognition, identification, and classification,^130^ vision and control systems, cancer predictions,^131^*etc.*). Recently, machine learning has emerged as an attractive tool to reduce model complexity.^132^ As a subset of machine learning, ANNs have been successfully used for a variety of applications,^133^ some including structural analysis^134^ and material science, ranging from studies of atomic properties^135^ to the mechanical properties of individual CNTs.^136^

### Architecture of an ANN

**Fig. 3:**
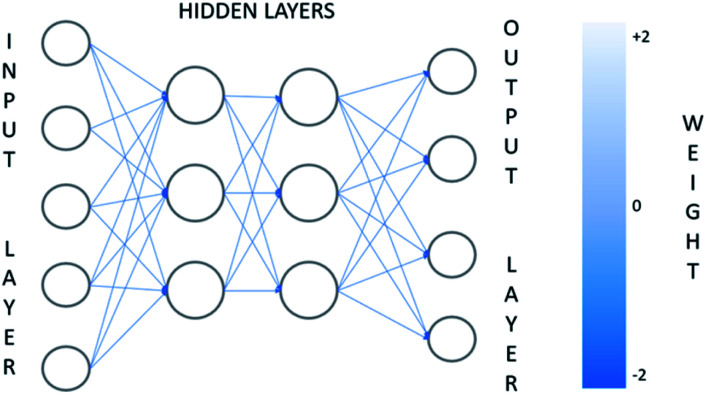
Schematic of the architecture of an ANN.

The neural network approach is a way of modeling data based on computer learning in order to perform complex functions. The ANNs are composed of simple elements operating in parallel, adaptive and distributed systems. In most ANN implementations, the output of each artificial neuron is computed by some non-linear function of the weighted sum of its inputs. Artificial neurons, which are processing elements, are divided into multiple layers (Fig. 3), and their connections called edges, typically have assumed weights that are adjusting as the learning proceeds based on a technique called the back-propagation rule.^137^ The architecture of the ANN is formed by input and output layer and a series of hidden layers, each of which is formed by a finite number of neurons.^138^ This kind of ANN architecture is called a multi-layer network and is the most widely used. It should be noted that artificial neurons in some cases may have a threshold such that the signal is only sent if the total signal crosses that threshold. After building the database which is divided into subsets, the neural network is trained for a specific task needed.

The training phase makes it possible to calculate the synaptic weights leading to each formal neuron.^139^ The training of a network is a process where the set of adjusted weights is optimized to make the best prediction of the target variable on the basis of background variables. Multiple layer networks are trained with the standard back-propagation error algorithm.^137,139,140^ This algorithm presents training examples to the network, examining the shift between the network output and the desired output while modifying the weights and biases of connections until the network produces an output very close to the desired one.^139^ The back-propagation algorithm consists of a forward step where the signal propagates through the computational units until it gets to the output layer and a backward step where all the synaptic weights are adjusted accordingly to an error correction rule.^136^ There are some factors that affect the ability of ANNs to model problems such as network structure, training and testing dates, network algorithms and finally transfer, training, learning, and performance functions.^141^

### Applications of ANNs

ANNs offer fast solutions for a wide range of problems, especially the ones with real-time constraints.^139^ Currently, it's mostly used in the field of medicine for developing motor neurons^138^ or improving the monitoring of neural activity.^142^ Some works have been dedicated to predicting the sensitivity of strain sensing of piezoresistive materials, however computational cost can be considerably reduced by using machine learning.^143^

In terms of sensor systems, various problems regarding CNTs and their composites have been covered, processed or solved using an ANN approach. Menacer *et al.*^139^ developed a finite element model for an armchair CNTs using ANSYS simulator and examined their ability of detection of the applied force. It was found that the sensitivity is proportional to the nanotube length and that the non-linearity of the sensor depends on the applied force range. Moreover, this smart nanoforce sensor was proposed and investigated using ANN-based computation.^139^ Matos *et al.*^144^ made a FEM simulation of a CNT/polymer composite to produce training data for an ANN in the goal of developing a technique of precise tailoring of the electrical properties of composites. The authors conducted simulations of conductivity of different composites by adopting different conductivities of CNTs and the matrix, geometry of CNTs and their volume fractions of the composites. The objective was to reduce the number of problem parameters and it was concluded that their ANN model could facilitate other types of conductive composites.^144^ A further study conducted by the same authors included a non-uniform multiaxial strain field.^132^

Fakhrabadi *et al.*^141^ developed an ANN to predict the natural frequencies of CNTs with different types, lengths, and diameters and the effects of the attached masses on the first five natural frequencies of a special CNT. The well-known molecular mechanics and FE models are used to describe the vibrational behavior of this discrete structure before and after attaching a particle on it. The authors^141^ used MATLAB ANN toolbox to create a structure that consisted of several layers: one input, few hidden and one output layers. Hidden layers relate the input layer to output one by multiplying them to weight factors and adding some biases. First, the input data were inserted in ANN and the errors between real target values and the proposed target values by ANN were calculated. Next, the sensitivities start to propagate from the output layer toward the first layer and then the weights and biases are updated.^141^

Hayati *et al.*^137^ proposed an ANN for modeling and simulation of the semiconductive SWCNT transistor device. The proposed ANN model was optimized based on data obtained by using the MATLAB script.^137^ The application of machine learning implies very low computational cost and can be immediately used by relatively inexperienced users.^132^ ANN can be a powerful tool to model different problems, especially in cases that there are not accurate governing mathematical relations for them.^141^

#### ANNs in concrete mix design

The topic of using neural networks for predicting optimal concrete mix proportions has been explored many times in the past few decades. The research work^9–26^ included investigating the compressive strength and workability of plain concrete or concrete with different aggregates and additions. Probably the largest contribution in this area was given by Dr I.-C. Yeh which created the first database containing 1030 sets of data about properties of concrete specimens (mix proportions, age, compressive strength). The concrete specimens contained the additions of blast furnace slag, fly ash and superplasticizer in different proportions. The database was given by the UCI repository and has been used in research works since then.^11,14^

Some investigations have been directed toward specific types of concrete such as high-performance,^9,10^ green,^16,17,22^ self-compacting,^18^ and lightweight concrete. Khan *et al.*^15^ estimated the compressive strength of plain concrete using MATLAB ANN simulation and after comparison of the mathematical results and 55 experimental results, concluded that multilayer feed-forward neural network gives satisfactory results. Although the multilayer feed-forward approach is mostly used,^9,10,14,17,21,24^ researchers often use other techniques for predicting the compressive strength of concrete, such as fuzzy logic, decision tree or support vector machine.

The study carried out by Ziolkowski *et al.*^23^ showed how ANNs can be applied in civil engineering practice. The authors designed the optimal ANN architecture and fed it with an extensive database including 741 concrete mix recipe record and a corresponding laboratory destructive test. The final product was a system for obtaining optimal concrete mixtures which can be used by unexperienced users in everyday practice. Some authors showed the efficiency of ANNs by comparing the results from the ANN model with the results from fuzzy logic model,^20^ multiple linear regression model,^25^ random forest model,^21^ decision tree,^21^ support vector machine,^24^*etc.*

## Structural health monitoring systems

Civil engineering structures are generally the most expensive investments, with concrete (cement) as the second most widely used material in the world. Civil and industrial structures are constantly exposed to damage caused by the weather conditions in combination with poorly designed material properties, damage due to the over-usage of the structure, accidental occurrence, earthquake, *etc.* These damages manifest in the form of cracks, corrosion, displacement, delamination, rotation, strain and more, potentially resulting in a catastrophe if not regularly and properly monitored.

Because a civil structure should meet the criteria of safety, durability, serviceability, and sustainability during its service life, constant supervision over the condition of the structure is necessary. Structural assessment is done manually by visual inspection and in the intervals prescribed by the designer and/or regulations. These examinations are expensive, time-consuming and labor-intensive, as well as highly subjective and can only consider the damage that is visible on the surface of the structure.^71,145^ Having this in mind, the field of structural health monitoring (SHM) was developed in order to follow structural changes taking place under different environmental influences and loads.

The field of SHM has, since its inception, been developed in terms of sensor systems going from cabled to wireless, to MEMS (microelectromechanical systems) and ultimately developing NEMS (nanoelectromechanical systems).^124,146^ NEMS is currently the object of intense research activity in the field of detection, where the main objective is to minimize the MEMS surface and power consumption using carbon nanotubes.^139,147,148^ Nevertheless, more advanced and accurate methods for structural health monitoring are available and continue to be developed.^36,149^

More advanced methods typically involve the analysis of measurements from sensors such as displacement transducers,^40^ accelerometers and strain gauges,^150^ which provide real-time information that may be continuously monitored from a central location and give insight into the condition of the structure.^36,145^ One of the main limitations of sensors is that they are discrete fixed-point directional sensors, not being a part of the material or structure that is being monitored. This on occasion leads to discrepancies in readings, showing the incompatibility between the sensor and the monitored element. The need to develop sensors that can be a part of the material and used for multidirectional and multiple location sensing is, therefore, imposed.^79^ Self-sensing CNT/concrete composite is considered a good candidate for solving the durability and incompatibility issue between conventional sensors and concrete structures because it itself functions as both the structure and the sensor.^41,92,151^ With the development of smart, self-sensing materials including CNT/concrete composite which provide an electrical response to stress or strain,^36,56,152^ self-monitoring structures can become a civil engineering practice.

### Smart materials

Smart materials are engineered intelligent materials with properties adaptable to external stimuli (stress, moisture, temperature, light, acidity, radiation, *etc.*) in a controlled fashion. These composite materials are usually designed as a compound of the main insulating material and a filler material which gives it multifunctionality. Within the general field of civil engineering, structural design and SHM are the areas which mostly emplore these materials. Multifunctionality is introduced to structures exposed to severe loading, such as skyscrapers, bridges, structures in seismically active areas and aesthetically important structures. Saafi^97^ presented how the use of CNTs has opened the door for new smart and advanced sensing materials that could effectively be used in structural health monitoring where wireless and real-time sensing^72,145^ could provide information on damage occurrence and progression. CNTs have exhibited extraordinary mechanical properties, providing structural and functional capabilities simultaneously, including actuation,^52,153^ sensing, and generating power. These abilities represent the possibility for developing actuators capable of high stress and strain operating at low voltage, and multi-functional electrochemical and mechanical sensors.^34^

Piezoresistivity is defined as the change of electrical resistivity under the applied strain, and in order to obtain a piezoresistive cement-based material, an electrically conductive material such as carbon nanotubes has to be incorporated into a cementitious matrix.^124,154^ The piezoresistive behavior of CNT networks was first presented with CNT/polymer composites, whereupon application of mechanical load, the configuration of the networks was affected resulting in a change of the electrical resistance.^36,38,40,98,104^

Zhu^43^ concluded that piezoresistivity can be significantly influenced by the CNT types, aspect ratio, intrinsic electrical conductivity and the dispersion condition that occurs in the processing and curing stages. Kang *et al.*^37^ presented an examination of a polymer film strain sensor that was cast and bonded onto a glass fiber beam in order to test the macroscale strain sensing characteristics. The beam displacement and change of resistance of the sensor were simultaneously measured to build a strain response model and to find the sensitivity of the sensor. Because of their piezoresistivity, carbon-based structures have attracted special attention in the field of high-performance nanomechanical sensors^43,56,101,155^ and numerous studies in the range of experimental and computational methods have been carried out.^72,156–162^

### State-of-the-art in monitoring systems

The authors^107^ conducted a detailed parametric study and proposed an approximate simplified model for smart concrete strain sensors that are easy to handle in practical full-scale applications. The configuration of CNTs to aligned fibers is recognized as a natural embodiment for a one-dimensional material and the most effective to transfer axial properties to the macroscale.^54,78^

Carbon nanotube networks are highly valuable in SHM for strain mapping, damage detection and identification of crack initiation and propagation.^54^ Abot *et al.*^92^ produced a thread spun from carbon nanotube forests by growing and drawning CNT forests that were twisted into a fine thread (or yarn) in order to be used as the sensor element. The authors proved^92^ that this CNT thread is suitable as a distributed sensor material, allowing the development of a robust and efficient SHM system.

Kang *et al.*^37^ proposed a new approach for developing smart composite material able to monitor its own health while the actuation capability allows it to actively improve the performance of the structure and extend its life. The high strength and elastic modulus, as well as piezoresistivity, indicate the possibility to produce a long continuous sensor that would be able to measure strain over a large surface area for SHM of concrete structures. The authors^37^ developed a long continuous strain sensor (structural neuron) as an element in a biomimetic artificial neural system used for SHM, which is capable of measuring large strain and forming a grid over a large area of a structure, and on the other hand, simultaneously has a load-carrying capability as a structural component.

Fabrication of CNT neurons as long films has been proposed by some authors.^163,164^ The artificial neural system is primarily developed within the medical field where the application of CNTs includes electrical interfaces for neuronal stimulation and recording,^29^ neural prosthetic devices for artificially restoring impaired neural function,^165^ encouraging neuronal growth,^28^ retinal implants,^165^*etc.* In a functioning nervous tissue, neurons carry electrical signals *via* switching of the membrane potential caused by electrically and chemically gated ion channels in the cell membrane. Although the signal propagating along the length of the neuron is primarily achieved electrically, neurons communicate with each other at chemical junctions, synapses,^142^ much so like the communication between CNTs by electron hopping at their junctions. The same principle constitutes the artificial neural system which uses multiple neurons for real-time monitoring of strain of the structure. Since the production of neurons involves a mask to define the pattern, it can take any shape including a grid that resembles that of the peripheral neural system of the human body. Sensors act as nerves and actuators act as muscles where the control center is the brain.

The idea of a self-sensing structure is to mimic a biological system through sensing, actuation, adaptability, self-repair, *etc.* and the ultimate form of intelligent structures are the ones that have the additional ability to learn in contrast to the pre-programmed response. This learning feature is realized by employing artificial intelligence, namely machine learning and vis-à-vis artificial neural networks.^145,166^ The choice of ANNs to model so many different systems is, in part due to their flexibility, adaptability and generalization capabilities and their easy application in software and hardware devices^138^ and materials.^144^

### Proposal for application of ANNs and CNT/concrete composites in structural health monitoring

The authors of this paper propose a novel SHM system that would imply concepts of self-sensing concrete containing an artificial neuron system in combination with artificial neural networks. Real-time effective and long-distance monitoring of concrete structures is possible to obtain by developing a cementitious material representing a good host for the intricate branches of long continuous CNT neurons on one side and on the other – trained neural network capable of disregarding negligible data and processing relevant information about the structure's condition, so it may generate a 3D model showing possible damage and current state of structural elements. In the case of the maintenance planning of a new structure, ANN could also be used to obtain the optimal mixture of CNT/concrete composite in terms of desired specific properties of the end product such as *e.g.* high permeability, assigned porosity, high flexure, self-cleaning ability, *etc.*

## Conclusions

This paper reviews previous research work which is exploring the properties of CNTs and their influence on concrete, and the use of artificial neural networks in material science and concrete technology. The paper shows how incorporating carbon nanotubes in concrete mixtures or alternatively grafting short CNTs to produce excessively long carbon fibers, combined with the application of precisely trained ANNs represents a new direction in the evolution of structural health monitoring of concrete structures. The capability to tailor materials at the nanoscale has opened-up a new field of research aimed at both tailoring their properties as well as developing intelligent systems that are capable of sensing and actuation. Several studies were conducted on the topic of different types of strain sensors^79,150,167^ made with nanomaterials as fillers. The goal is not only higher strength of the composite material, but also obtaining multifunctional smart and cost-effective structures. The behavior of CNT-based sensors and actuators involves multiple phenomena at length scale hierarchies ranging from quantum to macroscopic scale. Although there are still some challenges regarding the proper dispersion^168^ of CNTs in the cement matrix and the exact volume fraction at the percolation threshold,^43^ the results of the work presented in this paper are very promising and indicative of the beginning of a new direction in the development of structural health monitoring. Fundamental knowledge of the structure/property relations of CNT composites is still somewhat lacking which highlights the need to develop both experimental and analytical techniques to bridge these scales toward the optimization of SHM systems.^49^ Nevertheless, smart nanomodified cementitious materials provide promising and innovative applications.^38^

Moreover, despite much work devoted to making a perfect ANN structure, there are no conclusive rules on how to achieve it. The only way to produce a suitable ANN structure includes the ‘trial and error’ process, so the number of hidden layers and the number of neurons at each layer is adjusted for each specific problem.^140^ Future investigation of the authors will focus on developing the methodology of production of self-sensing CNT/concrete composites and specifying, modeling and training the ANN by using the obtained results of sensitivity testing.

## Conflicts of interest

There are no conflicts to declare.

## Supplementary Material
